# The Diseases and Injuries of the Conjunctiva, Especially the So-called Granulated Lids

**Published:** 1899-01

**Authors:** 


THE DISEASES AND INJURIES OF THE CONJUNCTIVA, Especially the So-called Granulated Lids. By JOHN H. THOMPSON, M. D., Professor of Ophthalmology and Otology, Kansas City Medical College, Kansas City, Mo. First edition (with illustrations). Kansas City: Hudson Kimberley Publishing Co., 1014–1016 Wyandotte Street. 1897.

This little volume of 216 pages should be in the hands of every practitioner doing eye work. Nothing superfluous is written. No space taken up with the various theories. The diseases of the conjunctiva are given with the etiology, pathology, prognosis and treatment in a concise and lucid manner. Many will not agree with him on his theory of Bowman's membrane, definition of trachoma, or the statement that acute conjunctivitis is not directly contagious. The work is carefully and conscientiously written, scarcely anything of real practical bearing on the various diseases described being omitted, and many hints that could only come from a large experience are given.

